# Different Finite Durations of Anticoagulation and Outcomes following Idiopathic Venous Thromboembolism: A Meta-Analysis

**DOI:** 10.1155/2010/540386

**Published:** 2010-12-29

**Authors:** Aaron B. Holley, Christopher S. King, Jeffrey L. Jackson, Lisa K. Moores

**Affiliations:** ^1^Department of Pulmonary/Critical Care/Sleep Medicine, Walter Reed Army Medical Center, Pulmonary/Sleep and Critical Care Service, 6900 Georgia Avenue NW, Washington, DC 20307, USA; ^2^Department of Pulmonary/Critical Care Medicine, William Beaumont Army Medical Center, 5005 N. Piedras Street, El Paso, TX 79930, USA; ^3^Department of Medicine, The Uniformed Services University of the Health Sciences, 4301 Jones Bridge Rd, Bethesda, MD 20814, USA

## Abstract

*Introduction*. Controversy remains over the optimal length of anticoagulation following idiopathic venous thromboembolism. We sought to determine if a longer, finite course of anticoagulation offered additional benefit over a short course in the initial treatment of the first episode of idiopathic venous thromboembolism. *Data Extraction*. Rates of deep venous thrombosis, pulmonary embolism, combined venous thromboembolism, major bleeding, and mortality were extracted from prospective trials enrolling patients with first time, idiopathic venous thromboembolism. Data was pooled using random effects meta-regression. *Results*. Ten trials, with a total of 3225 patients, met inclusion criteria. For each additional month of initial anticoagulation, once therapy was stopped, recurrent venous thromboembolism (0.03 (95% CI: −0.28 to 0.35); *P* = .24), mortality (−0.10 (95% CI: −0.24 to 0.04); *P* = .15), and major bleeding (−0.01 (95% CI: −0.05 to 0.02); *P* = .44) rates measured in percent per patient years, did not significantly change. Conclusions: Patients with an initial idiopathic venous thromboembolism should be treated with 3 to 6 months of secondary prophylaxis with vitamin K antagonists. At that time, a decision between continuing with indefinite therapy can be made, but there is no benefit to a longer (but finite) course of therapy.

## 1. Introduction

Pulmonary embolism (PE) and deep venous thrombosis (DVT) are thought to be part of the same disease process, and together they are referred to as venous thromboembolism (VTE). In the general population, VTE occurs in 1-2 out of every 1000 people per year or 0.1% per person per year [1]. Anticoagulation is the primary treatment for this disease, and efficacy is well established [2]. 

The prescribed duration of anticoagulation is dependent on the risk for recurrent thromboembolic events. Good evidence supports limited duration treatment for patients with transient risk factors [2]. For the first episode of idiopathic (unprovoked) VTE, controversy remains over the appropriate length of therapy [1–4]. The Seventh edition of the American College of Chest Physicians Guidelines [5] on antithrombotic therapy recommended at least 6 to twelve months of anticoagulation for patients with a first idiopathic VTE event, with consideration of indefinite therapy. These recommendations were based upon the principle that secondary prophylaxis is effective (less than 1% recurrence/year while on therapy) [3] and patients with idiopathic events are more likely to suffer recurrences after anticoagulation is discontinued [4, 6, 7].

Several systematic reviews have attempted to address length of therapy following VTE, but none have focused exclusively on comparing finite durations following an idiopathic event [8–11]. In 2008, the 8th American College of Chest Physicians (ACCP) consensus guidelines updated treatment recommendations for the first episode of idiopathic VTE [2] by modifying their previous recommendation of 6–12 months treatment [5], to “at least three months.” This change reflects the uncertainty surrounding the appropriate duration for anticoagulation [12, 13]. 

The guidelines also recommend individualized risk stratification and consideration for life-long therapy. Although tools to estimate risk for VTE recurrence are available, they have not been well validated in management or outcome studies [14, 15]. For the individual patient, balancing bleeding with recurrence risk remains difficult [4]. Until individualized risk stratification can be done in a systematic, reliable, and safe manner, physicians will need to decide between finite or life-long anticoagulation in these patients. Because physicians and patients may opt against life-long therapy for the first idiopathic VTE occurrence, we sought to define the safest and most effective duration for finite therapy. Our goal was to pool studies evaluating treatment duration following idiopathic VTE, excluding all patients with transient or identifiable permanent risk factors. We focused primarily on VTE recurrence rates after anticoagulation is discontinued.

## 2. Methods

### 2.1. Literature Search

Two investigators independently searched the published literature (1964 through January 2009) for prospective cohorts and randomized controlled trials (RCTs) evaluating oral anticoagulation for the first episode of idiopathic VTE. The search was not limited to the English language. Databases included were Medline, EMBASE, http://ClinicalTrials.gov/, Computer Retrieval of Information on Scientific Projects, Cochrane Controlled Trials Registry, ACP Journal Club, Cochrane Database of Systematic Reviews, and Databases of Abstracts and Reviews of Effectiveness. Search terms were “deep venous thrombosis,” “pulmonary embolism,” and “venous thromboembolism.” Hand searching of cited bibliographies was performed for completeness.

### 2.2. Study Selection Criteria

Inclusion criteria were as follows: (1) RCT or prospective cohort study enrolling patients with an initial episode of idiopathic VTE, (2) documented duration of anticoagulation of at least three months, (3) documented duration of follow-up postanticoagulation, (4) monitoring of adverse events, to include major bleeding, recurrent VTE, and death, and (5) objective confirmation of initial and recurrent DVT (Doppler ultrasonography, impendence plethysmography, radiofibrinogen uptake scanning, venography) or PE (computed tomography (CT) angiogram, pulmonary angiogram, ventilation perfusion scanning). 

Studies that included pregnant patients or patients less than 18 years old were excluded. Data from patients with prior episodes of VTE or transient risk factors for VTE were excluded from the analysis. Patients who had known malignancy, antiphospholipid antibody syndrome (APAS), antithrombin III (ATIII) deficiency, or protein C or S deficiency diagnosed at the time of enrollment were excluded from the analysis. Patients diagnosed with the Factor V Leiden or prothrombin 20210A mutation were not excluded. 

For studies that enrolled patients with both first time, idiopathic VTE (inclusion criteria) and those with transient or permanent risk factors, or previous VTE (exclusion criteria), attempts were made to separate outcomes data. When this was not possible, the primary author was contacted via email for additional information. If no response was received within 3 weeks, a second email was sent. If the author could not be reached and it was not possible to separate outcomes data, the study was excluded. Because most studies did not systematically screen for cancer or thrombophilia at enrollment, these conditions were occasionally diagnosed and reported during study follow-up. The patients diagnosed with cancer or thrombophilia during follow-up were not excluded. Two authors (Christopher King and Aaron B. Holley) independently selected articles for inclusion. Disputes regarding inclusion were presented to a third party (Lisa K. Moores) and then resolved by consensus.

### 2.3. Study Quality Assessment

All studies were rated independently for quality by two investigators (CSK and ABH) using the index of Jadad et al. [16] for RCTs (Table 1). There was 100% agreement regarding quality ratings on first review.

### 2.4. Data Extraction

The following data were extracted from each article: mean/median age; sex distribution; mean/median duration of anticoagulation; mean/median duration of follow-up following cessation of anticoagulation; DVT, PE, and combined VTE recurrences during and after anticoagulation; major bleeding during and after anticoagulation; and mortality rate. When bleeding data were not available but authors were contacted, we requested they use the following definition for major bleeding: clinically overt and associated with either a decrease in Hgb ≥ 2 g/dL or transfusion of ≥2 units pRBCs, if it was retroperitoneal or intracranial, or if it warranted the permanent discontinuation of the study drug. Fatal bleeding events were included in the mortality rate.

Data were extracted independently by two investigators (CSK and ABH); disagreements were settled by consensus. Primary outcomes were VTE (DVT and/or PE) recurrence and mortality rate, percent per patient years, after treatment cessation. We also determined the rate of major bleeding both on and off therapy. As a secondary outcome we created an adverse event rate consisting of the rate of VTE recurrence plus major bleeding events off of therapy.

### 2.5. Statistics

All event rates were converted to percent per patient year to adjust for variations in duration of follow-up after cessation of therapy. Data were pooled using random-effects methods. For metaregression modeling, a restricted maximum likelihood estimator was used [17]. Sensitivity analyses were done both by stratifying on the indicator variable and by including the term as an additional independent variable in regression modeling.

We analyzed all outcomes as continuous variables based on increasing duration of treatment. We also combined studies into three different groups based on whether they initially received 3–6, 6–12, or >12 months of treatment. These groups were chosen because they are commonly recommended in guidelines and studied in trials and are therefore clinically relevant. Outcomes were compared across groups, and when significance was found, pairwise comparisons were performed to determine where the difference occurred.

## 3. Results

**Figure 1:**
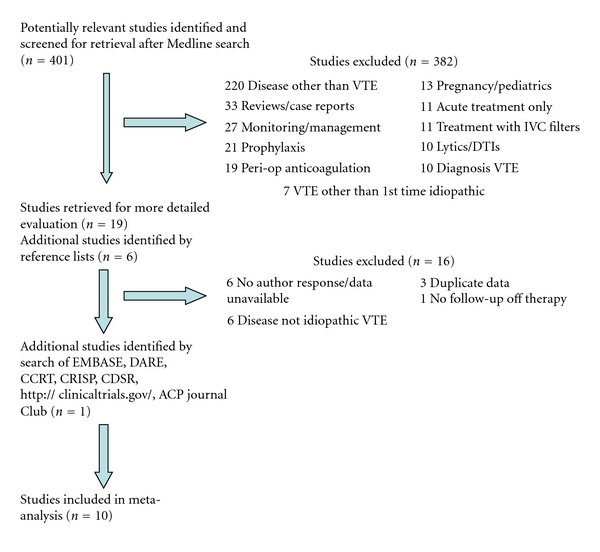


The initial search yielded 401 studies. Of these, 382 were excluded, mainly because they dealt with disease processes other than VTE (Figure 1). Ultimately, 10 studies, with a total of 3225 patients, met our inclusion criteria (Table 2). Two studies were combined because one [18] reported extended follow-up data on the same patient group enrolled in the other [19]. The average duration of treatment was 9.7 ± 5.5 months and the average length of follow-up off of treatment was 27.6 ± 9.1 months. 

There were 6 RCTs [6, 7, 19–21] and 4 prospective cohorts [22–25] studies, with 1306 and 1919 patients, respectively. Of the RCTs, 4 had two arms receiving different durations of therapy that were both eligible for inclusion (first time idiopathic VTE with documented follow-up off of therapy) [6, 7, 19, 20]. The study by Palareti et al. [19] consisted of two groups, one with patients that were negative for D-dimer at the end of treatment and the other with patients who were D-dimer positive. The positive D-dimer group had patients randomized to continued anticoagulation or cessation of treatment. We included the D-dimer negative group and the D-dimer positive group that was randomized to treatment cessation in our analysis. The D-dimer positive group that was randomized to continue anti-coagulation had no documented follow-up off of therapy and was therefore excluded. This group published extended follow-up on their patients in 2009, and these data were included as part of our analysis [18]. Lastly, one RCT had only one arm eligible for inclusion, with the second arm excluded because patients were treated for only 6 weeks [21]. 

Overall age and percentage male, both of which would be expected to affect the VTE recurrence rate, are listed in Table 2. Of note, the trial by Kyrle et al. [22] separated groups by gender and showed a significant increase in recurrences for males.  Table 3 describes additional differences that might be expected to affect outcomes. Relevant patient population criteria are described, such as whether patients with DVT and PE were enrolled and the percent time that INR was in the target range during treatment. The study by Kyrle et al. [22] was the only one to document hormone replacement (HRT) and oral contraceptive (OCP) use. A few studies screened for thrombophilia prior to enrollment [19, 21–23] but most simply excluded patients with known disease. No studies screened for cancer, but four studies [6, 7, 19, 22] reported cancer diagnoses during follow-up.

Table 3 also lists the frequency of follow-up visits. For all studies, patients were specifically instructed to contact investigators, or their primary doctors, for signs or symptoms of recurrent VTE. The percentage of recurrent events that were idiopathic is also listed in Table 3. 

### 3.1. VTE Recurrence Rates

Table 4 lists the absolute number of recurrences, the duration of follow-up, and the VTE event rate in percent per patient year after therapy was stopped. Several studies did not provide VTE events broken down separately into DVT and PE [6, 7, 21, 22]. The majority of studies, regardless of therapy duration prior to cessation, had VTE recurrence rates between 4.6 and 8.8% per patient year. The 120 patients in the Cosmi et al. [18] and Palareti et al. [19] study who were D-dimer positive but had treatment withheld had a slightly higher rate of 9.6% per patient year, while the all female arm of the Kyrle et al. [22] study had a lower than average rate of 2.0% per patient year. One arm of the Farraj [20] study had only 12 months of follow-up off treatment and the Palareti cohort [25] provided 16 months. All other studies had >22 months of follow-up off of anti-coagulation.

**Figure 2:**
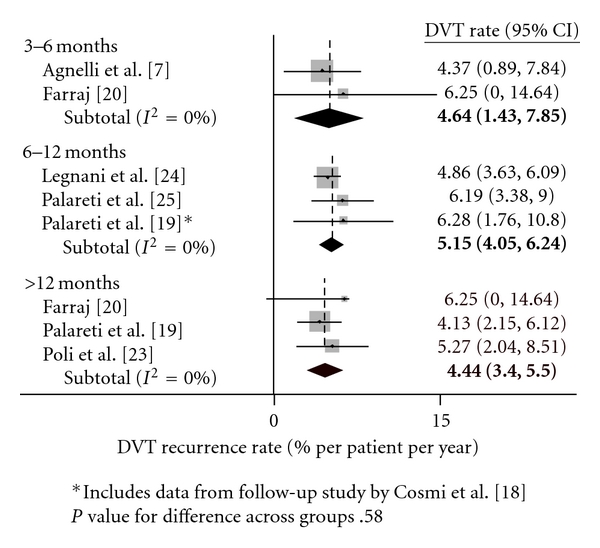
DVT recurrence rates after treatment cessation.

**Figure 3:**
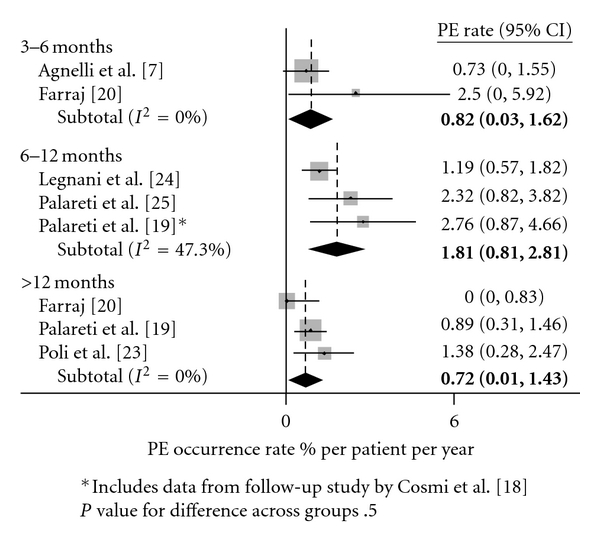
PE recurrence rates after treatment cessation.

**Figure 4:**
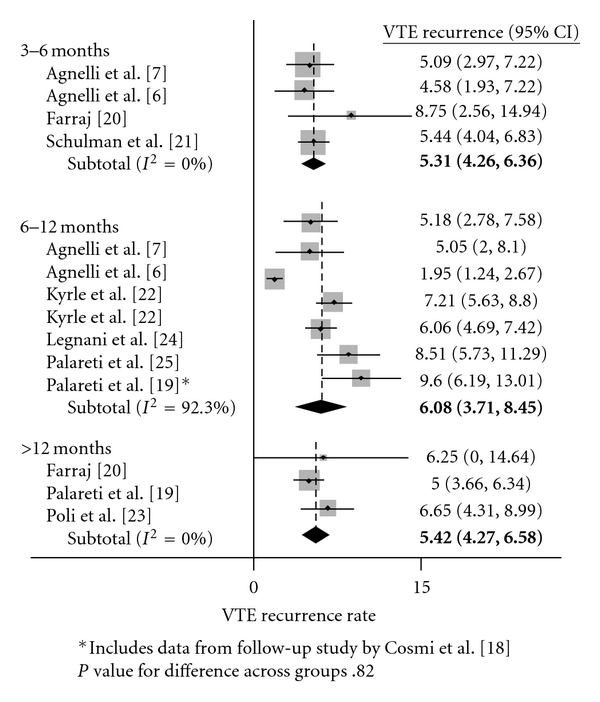
VTE recurrence rates after treatment cessation.

Table 5 lists the change in recurrence rates for each additional month of anti-coagulation prior to cessation. For increasing durations of therapy, there was no significant change in DVT, PE, and VTE rates as measured in percent per patient years. Figures 2, 3, and 4 show recurrence rates off of therapy in peto-plots, based on whether patients initially received 3–6, 6–12, or >12 months of therapy. Pairwise comparisons between groups were also performed, and no significant differences in recurrence rates were found.

### 3.2. Bleeding Rates

Table 4 lists bleeding rates off therapy. Generally, bleeding was considered “major” if it was clinically overt and associated with either a decrease in Hgb ≥ 2 g/dL or transfusion of ≥2 units pRBCs, if it was retroperitoneal or intracranial,or if it warranted the permanent discontinuation of the study drug [6, 7]. Farraj [20], Palareti et al. [19], and Cosmi et al. [18] used the same definition but did not include “bleeds warranting permanent discontinuation of the study drug.” Schulman et al. [21] defined major as “any bleeding requiring hospitalization, treatment with vitamin K, treatment with blood, or both.” Neither Kyrle et al. [22], Poli et al. [23], nor Palareti et al. [25] systematically assessed for bleeds, but via correspondence all three confirmed that no major bleeds (according to our predetermined definition) were recorded after therapy was stopped.

Several factors might be expected to alter bleeding rates across studies. Some investigators used the presence of a previous bleed on anticoagulation as part of their exclusion criteria, which would bias these studies toward having fewer bleeding events than the others. Although only the two Agnelli et al. [6, 7] studies specifically stated that they excluded such patients, one would assume that any study that randomized patients to extended treatment after an initial period of therapy would have done the same for ethical reasons [19]. Target INR in the Schulman et al. [21] study was only 2.0–2.85, while all others targeted 2.0-3.0. Some studies included patients on acenocoumarol [6, 7] or dicumarol [21] as well as Coumadin. The lack of formal assessment for bleeding in the Kyrle et al. [22], Poli et al. [23], and Palareti et al. [25] studies means that their results likely underestimated bleeding.

Most studies enrolled patients after treatment had already been stopped, and only 4 had data available for bleeding rates while on anti-coagulation [6, 7, 20, 21]. Very few bleeds off of therapy were recorded, which is consistent with a lack of persistent elevation in bleeding risk once anti-coagulation is stopped. Table 5 lists the change in bleeding rates both on, when available, and off of therapy. As was the case with thromboembolic recurrence rates, there was no significant change in the bleeding rate, as measured in percent per patient years, with each additional month of anti-coagulation, regardless of whether the patient was on or off anticoagulation.

### 3.3. Adverse Events Combined

Only 4 studies had data for bleeding and recurrence rates, both on and off of therapy [6, 7, 20, 21]. This made our data difficult to analyze in terms of balancing the excess bleeding expected from extending therapy with any possible benefit in reducing VTE once therapy is stopped. Both of the Agnelli et al. [6, 7] studies found that longer durations increased the number of bleeds but did not reduce VTE rate at two years. The Farraj [20] study did show a benefit for 24 versus 6 months of anti-coagulation, with recurrence rates of 6.3% versus 8.8% per patient year, off of therapy, respectively (Table 4). Each group suffered 2 bleeds while on therapy, and no bleeds off of therapy. This study had fewer patients and a lower Jadad score when compared to the Agnelli et al. studies (Table 2), and the arm that received 24 months of anti-coagulation had only 12 months of follow-up recorded off of therapy. The Schulman et al. study compared 6 months to 6 weeks of therapy, and only patients in the 6 month arm met our inclusion criteria. There were 3 bleeds while on therapy in the 6 month arm (2.1% per patient year) [21].

Otherwise, analysis of the combined outcome of bleeding and VTE recurrence off of therapy was limited by the small number of bleeds. Comparing the combined outcome was essentially a repeat assessment of the VTE recurrence rate across studies.

### 3.4. Mortality

Following treatment cessation, we found no statistically significant decrease in mortality rate with extension of initial therapy duration (Table 5). Most studies were specific in attributing death to either bleeding, VTE, or another cause. However, it was not clear how long each patient was treated with anti-coagulation prior to death, and whether or not they were on therapy when they died. For the Palareti et al. [25] and Kyrle et al. [22] studies, no mortality data is listed because deaths could not reliably be assigned to a specific group (Table 4).

**Figure 5:**
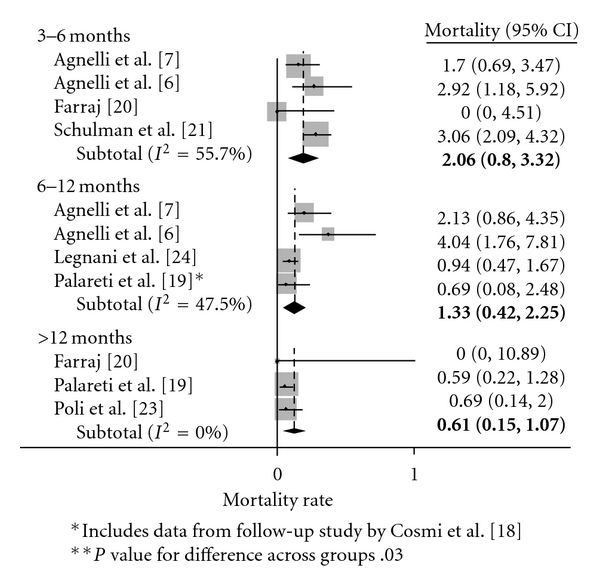
Mortality rate

Figure 5 displays mortality differences in a Peto-plot, based on whether patients initially received 3–6, 6–12, or >12 months of therapy. The difference in mortality across groups was statistically significant. In pairwise comparisons, there was a trend toward a significant decrease when the >12 was compared with the 3–6 month group (*P* = .05), but no difference when the >12 was compared to the 6–12 month group (*P* = .32).

### 3.5. Additional Analyses

We reanalyzed our data looking at cohort studies versus RCTs, studies with Jadad ≤4 versus >4, cohort age, and the percentage of each cohort that was male. For all outcome events, changes in rates with extensions of therapy were not statistically significant. 

Two additional RCTs that enrolled patients with idiopathic VTE but met exclusion criteria, one because several patients in one arm were diagnosed with APAS at enrollment [26] and the other because one third of patients had previous VTE [27], were added back to see if their inclusion would impact our results. For each additional month of anticoagulation, there was still no statistically significant decrease in DVT, PE, VTE, or major bleeding rates after therapy was stopped. There was a trend toward reduced mortality with longer durations of treatment (*P* = .09). When duration of therapy was categorized in 3 different groups, 3–6, 6–12, and >12 months, there were no significant differences in DVT, PE, VTE, or major bleeding rates. Pairwise comparisons for mortality again showed significantly lower rates for patients treated >12 months in comparison to 3–6, but not to 6–12 months.

## 4. Discussion

Controversy surrounds the optimal duration of secondary prophylaxis with anticoagulation in patients with an initial idiopathic event. Studies over the past decade have been consistent in the finding that therapy is effective, and recurrent VTE is rare while on anticoagulation. However, anywhere from 8–20% of patients will experience a recurrent event within 10 years of stopping anticoagulation [6, 12, 21]. Recurrence rates depend on patient-related factors surrounding the initial event. Patients whose initial event is idiopathic have a higher rate of recurrence, [6, 28] which makes this a population of great interest.

Several systematic reviews have attempted to address length of therapy following VTE, but none have focused exclusively on comparing finite durations following an idiopathic event [8–11]. Ours is the first systematic review and quantitative assessment to do so. We have found that recurrence rates after discontinuation of anticoagulation are not dependent on the duration of the initial course of therapy. There is no statistically or clinically significant decrease in recurrence with longer (but finite) courses of anticoagulation. 

To balance the benefits and risks of extending anticoagulation, we attempted to capture major bleeding rates, and an adverse event rate defined as a combination of recurrent VTE and bleeding. Unfortunately, only three of the included studies [6, 7, 20] allow any direct comparison of bleeding rates both on and off of therapy in two separate arms, making it difficult to quantitatively assess the balance between excess bleeding risk and recurrent VTE for a given duration of treatment. Therefore, the focus of our review was on outcomes after discontinuation of therapy, and bleeding rates were expectedly low during this period. Still, it is clear from the Agnelli et al. [6, 7] and Schulman et al. [21] studies that extended durations increase the absolute number of bleeds. Because we could not find a benefit in VTE reduction with finite durations greater than 3 to 6 months, the excess bleeding risk during extended, finite therapy poses unnecessary risk.

Our study suffers from several limitations. There is significant heterogeneity among studies included in this analysis. The differences in baseline patient risk factors that are outlined in Table 3 account for some of the rate differences across studies. We attempted to address this by using a random effects model and by performing several sensitivity analyses. In addition, we used a mean duration of initial treatment for each study, rather than trying to obtain patient level data. This assumes that the mean duration applies to all patients in the study, when, in reality, several of the studies [19, 24, 25] included significant numbers of patients that would have fallen into each category — 3–6, 6–12, and >12 months. Finally, if we were unable to contact authors for information that would separate outcomes from provoked or unprovoked, the study was excluded. We cannot exclude the possibility that this data may have altered the final analysis. Our study is also limited by the same shortcomings that may affect all meta-analyses. Publication bias may have limited data on studies where outcome did not differ between different durations of anticoagulation. There is also the possibility that relevant studies were not identified, although we feel that the likelihood of this was low as the search was independently performed by two investigators. 

Mortality estimates must be viewed with caution as the overall numbers were small and it was often difficult to determine treatment duration and whether deaths occurred on or off therapy. For the studies in which cause of death could be definitively determined and assigned to a specific group, only a fraction of the total was due to VTE or major bleeding: Agnelli et al. 2001 [7]: 0/14; Legnani et al. 2006 [24]: 2/11; Palareti et al. 2006 [19]: 1/8; Kearon et al. 1999: 1/3 [26]; Ridker et al.: 3/8 [27].

The Palereti et al. [19] trial may have biased the results in favor of a longer duration of therapy. For the purposes of our analysis, the Palareti et al. [19] study was broken up into two arms, one treated for 10.8 months and consisting of patients positive for D-dimer, and one treated for 13.0 months and consisting of patients negative for D-dimer (Table 4). Because positive D-dimer is a recognized marker for increased VTE recurrence risk [29], including this study would favor >12 months over <12 months of treatment, even though this difference is more likely due to patient selection than treatment duration. 

The short duration of follow-up in the 24-month treatment arm of the Farraj [20] study may also have biased results. The 12 months of follow-up provided for this arm were significantly less than the average across included studies (27.6 ± 9.1 months). Immediately following treatment cessation the VTE recurrence rate usually increases [6, 7, 9, 10] and then tapers off over time. For example, Agnelli found that two thirds of all VTE recurrences occur in the first year after stopping anti-coagulation [7]. Follow-up beyond 12 months may have decreased the recurrence rate for this arm. Otherwise, the long average duration of follow-up off therapy for studies in our analysis is a definite strength.

In conclusion, our analysis confirms that across the broad spectrum of patients considered to have idiopathic VTE, extending anti-coagulation beyond three to six months will not decrease recurrence risk after therapy is stopped. As our ability to tailor therapy based on individualized risk factors improves, finite durations for specific groups may be identified. Until then, a general bleeding and VTE recurrence risk assessment should occur after the initial 3 to 6 months of anticoagulation, with the understanding that extended but less than life-long anti-coagulation may increase the short-term bleeding risk without decreasing the long-term VTE recurrence rate.

## Figures and Tables

**Table 1:** 

Study described as randomized
Appropriate randomization method (i.e., random numbers table)
Study described as double blind
Appropriate blinding (i.e., identical placebo)
Description of withdrawal and dropouts
Methods of statistical analysis described
Clear description of inclusion and exclusion criteria
Description of methods to assess adverse effects

**Table 2:** Characteristics of included studies.

Trial	Design	Patients	Age (mean)	%male	Tx Duration (mos)	Jadad Score	Jadad Problems
Agnelli et al. [6]	RCT	90	—	—	12	6	Not double blind
		91	—	—	3		
Agnelli et al. [7]	RCT	134	67	55	12	6	Not double blind
		133	68	61	3		
Farraj [20]	RCT	32	41	62	24	4	Not double blind Withdrawals/dropouts No statistics methods
		32	42	56	6		
Legnani et al. [24]	Cohort	628	67	53	7	4	Not randomized
Kyrle et al. [22]	Cohort	453	45	0	9	4	Not randomized
		373	51	100	8		
Palareti et al. [19]	RCT	385	59	55	13.0	6	Not double blind
		120	68	42	10.8		
Palareti et al. [25]	Cohort	282	70	55	7.5	4	Not randomized
Schulman et al. [21]	RCT	289	63	63	6	6	Not double blind
Poli et al. [23]	Cohort	183	60	57	14.9	3	Not randomized

**Table 3:** Qualitative analysis.

Trial	Design	Population	Follow-up (months)	Recurrence (% idiopathic)
Agnelli et al. [6]	RCT	PE, enrolled after 3 mos No cancer/thrombophilia screening INR 2-3 (83%) New cancer 11 patients	3, 6, 12 after randomization, then q6	84.8%
Agnelli et al. [7]	RCT	DVT, enrolled after 3 mos No cancer/thrombophilia screening INR 2-3 (81%) New cancer 5 patients	3, 6, 12 after randomization, then q6	100%
Farraj [20]	RCT	DVT and PE, enrolled after event No cancer/thrombophilia screening	q4 for 12 mos after cessation	100% 1 event on therapy (INR = 1.8, dose missed during travel)
Legnani et al. [24]	Cohort	DVT (71%), PE (29%) enrolled after 3 mos No cancer/thrombophilia screening 15 patients (2.4%) protein C, S def	3 mos after cessation, then q6 mos	86%
Kyrle et al. [22]	Cohort	DVT (58%) and PE (42%) enrolled after 3 mos No cancer screening, had systematic thrombophilia screening HRT/OCP not excluded (175 used OCPs, 61 on HRT), females encouraged to stop after VTE New cancer in 14 patients Distal and axillary DVT included	q3 mos for first year, then q6 mos	Not specified
Palareti et al.* [19]	RCT	Prox DVT (62%) and PE (38%) enrolled after 3 mos ATIII+APA screening, no cancer screen New cancer in 13 patients	q3-6 mos intervals	Not specified
Palareti et al. [25]	Cohort	DVT and PE, enrolled after 3 mos No cancer/thrombophilia screening Distal DVT included D-dimer elevated in 49.3%	3 mos after cessation, then q6 mos	Not specified
Schulman et al. [21]	RCT	DVT (86%) and PE (14%), enrolled after event Prot C,S, ATIII screening, no cancer screen INR goal (2.0–2.85)	1.5, 3, 6, 9, 12, and 24 mos	Not specified
Poli et al. [23]	Cohort	DVT and PE, enrolled after 6 mos Prot C,S, ATIII, APA screening (only APA excluded), no cancer screen D-dimer elevated in 38%	Two in first year, one thereafter	97%

Enrolled after 3 months means that they were not recruited until after they had at least 3 months of therapy, as opposed to being recruited at the time of the initial event. All known cancer/thrombophilia was excluded; trials only got credit for systematic screening. INR range included for patients who had a period on therapy included. *Includes data from follow-up study by Cosmi et al. [18]

**Table 4:** Primary outcome events following cessation of therapy.

Trial	Patients	Tx (mos)	f/u (mos)	DVT	PE	VTE	Bleeding	Mortality	Adverse
Agnelli et al. [6]*	90	12	26.4	—	—	10 (5.1)	0 (0)	8 (3.1)	10 (5.1)
	91	3	31.7	—	—	11(4.6)	1 (0.4)	7 (2.9)	12 (5.0)
Agnelli et al. [7]*	134	12	29.4	—	—	17 (5.2)	0 (0)	7 (1.7)	17 (5.2)
	133	3	37.2	18 (4.4)	3 (0.7)	21 (5.1)	2 (0.5)	7 (1.7)	23 (5.6)
Farraj [20]	32	24	12	2 (6.3)	0 (0)	2 (6.3)	0 (0)	0 (0)	2 (6.3)
	32	6	30	5 (6.3)	2 (2.5)	7 (8.8)	0 (0)	0 (0)	7 (8.8)
Legnani et al. [24]	628	7	22.4	57 (5.5)	14 (1.3)	71 (6.8)	0 (0)	11 (1.1)	71 (6.8)
Kyrle et al. [22]*	453	9	38	—	—	28 (2.0)	0 (0)	—	28 (2.0)
	373	8	33	—	—	74 (7.2)	0 (0)	—	74 (7.2)
Palareti et al. [19]**	385	13.0	31.6	42 (4.1)	9 (0.9)	51 (5.0)	0 (0)	6 (0.6)	51 (5.0)
	120	10.8	28.7	20 (6.3)	8 (2.8)	28 (9.6)	0 (0)	2 (0.7)	28 (9.6)
Palareti et al. [25]	282	7.5	16.5	24 (6.2)	9 (2.3)	33 (8.5)	0 (0)	—	33 (8.5)
Schulman et al. [21]	289	6	42	—	—	55 (5.4)	0 (0)	31 (2.7)	55 (5.4)
Poli et al. [23]	183	14.9	28.6	23 (5.3)	6 (1.4)	29 (6.6)	0 (0)	3 (0.7)	29 (6.6)

( ): % per patient years, f/u is total, documented duration off of therapy; VTE: DVT and PE combined; Adverse Outcomes: VTE and major bleeding; Bleeding: see text for individual study definition of major bleeding

Major generally defined as clinically overt and associated with either a decrease Hgb ≥ 2 g/dL or transfusion ≥2 units pRBCs, if retroperitoneal or intracranial, or if warranted the permanent discontinuation of the study drug.

*VTE could not be separated into DVTs and PEs.

**Includes data from follow-up study by Cosmi et al. [18]

**Table 5:** Rate change (following cessation) for each additional month of initial anticoagulation.

Events	Rate (95% CI) ppy	*P*-value
DVT	−0.01 (−0.23 to 0.21)	.94
PE	−0.05 (−0.15 to 0.04)	.23
VTE	0.03 (−0.28 to 0.35)	.24
Bleed (on)	−0.23 (−1.22 to 0.75)	.75
Bleed (off)	−0.01 (−0.05 to 0.02)	.44
Deaths	−0.10 (−0.24 to 0.04)	.15

Rates measured in percent per patient year.
